# The Life Story Experience of "Migrant Dentists" in Australia: Potential Implications for Health Workforce Governance and International Cooperation

**DOI:** 10.15171/ijhpm.2016.135

**Published:** 2016-10-10

**Authors:** Madhan Balasubramanian, A. John Spencer, Stephanie D. Short, Keith Watkins, Sergio Chrisopoulos, David S. Brennan

**Affiliations:** ^1^Australian Research Centre for Population Oral Health, School of Dentistry, The University of Adelaide, Adelaide, SA, Australia.; ^2^Discipline of Behavioural and Social Sciences in Health, Faculty of Health Sciences, The University of Sydney, Sydney, NSW, Australia.; ^3^Australian Dental Council, Melbourne, VIC, Australia.

**Keywords:** Dentists, Workforce Governance, Health Policy, International Cooperation, Migration

## Abstract

**Background:** The migration of dentists is a major policy challenge facing both developing and developed countries. Dentists from over 120 countries migrate to Australia, and a large proportion are from developing countries. The aim of the study was to assess the life story experience (LSE) of migrant dentists in Australia, in order to address key policy challenges facing dentist migration.

**Methods:** A national survey of all migrant dentists resident in Australia was conducted in 2013. Migrant experiences were assessed through a suite of LSE scales, developed through a qualitative-quantitative study. Respondents rated experiences using a five-point Likert scale.

**Results:** A total of 1022 migrant dentists responded to the survey (response rate = 54.5%). LSE1 (health system and general lifestyle concerns in home country), LSE2 (appreciation towards Australian way of life) and LSE3 (settlement concerns in Australia) scales varied by migrant dentist groups, sex, and years since arrival to Australia (chi-square, *P* < .05). In a logistic regression model, migrants mainly from developing countries (ie, the examination pathway group) faced greater health system and general lifestyle concerns in their home countries (9.32; 3.51-24.72) and greater settlement challenges in Australia (5.39; 3.51-8.28), compared to migrants from well-developed countries, who obtained direct recognition of qualifications. Migrants also are more appreciative towards the Australian way of life if they had lived at least ten years in Australia (1.97; 1.27-3.05), compared to migrants who have lived for less than ten years.

**Conclusion:** Migrant dentists, mainly from developing countries, face challenges both in their home countries and in Australia. Our study offers evidence for multi-level health workforce governance and calls for greater consensus towards an international agenda to address dentist migration. Better integration of dentist migration with the mainstream health workforce governance is a viable and opportunistic way forward.

## Introduction

The migration of dentists is a major challenge affecting both the developing and well-developed countries. Dentists, similar to physicians and nurses, form a highly skilled group of health professionals involved in international migration.^1^ In a recent survey of all World Dental Federation (comprising of 200 national dental associations from over 130 countries) member countries, migration to the well-developed countries has emerged as a growing trend among dentists in developing countries.^2^ While migration appears to bring improved knowledge flows across borders and contribute towards global development,^3,4^ it can also lead to brain drain in poorer regions of the world.^5-7^

Australia is a popular destination for migrant dentists, trained from an overseas dental institution.^7,8^ Dentists from over 120 countries migrate to Australia,^9^ and it is estimated that one in four practising dentists in Australia is a migrant.^10^ A large proportion of migrant dentists arrive from countries with a similar historical and cultural proximity to Australia, such as New Zealand, the United Kingdom, Republic of Ireland, and Canada.^7,11^ Dentists from these countries can have their qualifications directly recognised for practise and can register fully as a dentist in Australia.^12,13^ A concern in recent years is the increase in the number of dentists arriving from developing countries, such as India, Philippines, South Africa, Indonesia, Egypt, Iran, and Iraq.^8,9^ These dentists are usually required to undergo a detailed examination process to gain registration to practice dentistry in Australia.^13^ Migrants can also choose to enter as students or academics and be associated with an Australian University, or obtain public sector employment in regional/remote areas in Australia with limited registration. The assessment and examination of overseas-qualified dentists is performed by the Australian Dental Council (ADC), which is a national accreditation authority overseeing dental education and training in Australia.^14^

The reasons for dentist migration are complex, and it is believed that a broad range of factors influence a dentist’s decision to migrate to Australia.^1^ Prior research suggests that dentists from the well-developed countries migrate to Australia for slightly different reasons (such as adventure) than dentists from developing countries, who migrate seeking better opportunities.^7^ Developing countries in South Asia and the Middle East are currently facing several health system challenges and political uncertainties that can affect dentists’ expectations, and thereby influence migration.^1^ Oral health systems in these countries seem largely unresponsive to dentist expectations on work conditions or better career opportunities.^15-18^ Migrant dentists arriving in Australia also face various settlement issues.^14,19^ The qualifying examination process for overseas-trained dentists is resource intensive, and can be challenging for newly arrived dentists, or migrants with a family to support.^14^ As suggested in other migration studies, migrants are also at the risk of discrimination, isolation, stress, and loss of identity in Australia.^20,21^ Migrant dentists face several cultural issues and require support for understanding the Australian way of life.^19^

To date, very little has been done to gain a thorough understanding of the migration and settlement experiences of migrant dentists in Australia. Prior studies were mainly qualitative^1,14,19^ and failed to provide any conclusive evidence. National dental labour force surveys^22-24^ dentist practice activity^25,26^ and job satisfaction^27,28^ studies in Australia have not presented information on migrant dentists. An understanding of their experiences both in home countries and in Australia can help uncover events that are likely to affect migrant dentists. Further, as experiences are unlikely to remain similar to all migrants, an assessment based on various migrant dentist characteristics, such as background and country of origin is vital. This will enable development of focussed and integrated migration policies into national or regional health workforce governance, and thereby support international cooperation. Therefore, the aim of the study was to assess the variation between migrant dentist (migration and settlement) experiences in function of migrant dentist characteristics in Australia.

## Methods

### Data Collection

A national survey of migrant dentists resident in Australia was conducted between January and May 2013.^7^ All migrant dentists registered with the Australian Dental Association (n = 1872) or enrolled as a graduate student in any of the nine dental schools (n = 105) in Australia were included in the survey. Participants were asked to fill a self-administered questionnaire; postal mailouts, handouts, and online approaches were used in the surveys. A wide variety of data including demographic, migration and residence characteristics, practice profiles, job satisfaction, and life story experience (LSE) were collected. Further details on study design, data collection, and data preparation procedures are described elsewhere.^7^ The focus of this study is limited to understanding the LSE of migrant dentists.

### Data Items

The LSE of migrant dentists was assessed through a battery of 38 items, comprising five empirical scales, designed through a qualitative-quantitative sequential study.^29^ These five scales provide necessary conceptual clarity and empirical grounding to explore migrant dentist experiences in Australia.^29^ Three scales (LSE1, LSE4, and LSE5) highlighted home country experiences of migrant dentists that led to migration into Australia; two scales (LSE2 and LSE3) captured settlement experiences in Australia. The list of all items included in each scale is presented in Supplementary file 1. “Health system and general lifestyle concerns” scale (LSE1), included 10 items that highlighted misgivings associated with migrant dentists home country health systems and living conditions. An example of an item from this scale is “*I was affected by corruption in my day to day practice in my home country*.” Two additional home country scales LSE4 (four items) and LSE 5 (five items) highlighted the attraction towards “society and culture” and “career development,” respectively. An example of an item from each of these two scales include *“I had a very active social life in my home country”* and *“I had very good mentors in my home country,”* respectively. “Appreciation towards Australian way of life” scale (LSE2), included 12 items and “settlement concerns” in Australia (LSE3) scale, had 7 items. An example of an item from the appreciation towards Australian way of life scale is: *“I like the cultural diversity in Australia”*; and an example from the scale settlement concerns in Australia is: *“I takes a lot of hard work to start a private practice in Australia.”* All survey participants were asked to specify their level of agreement with each LSE item. A five-point Likert scale was used to collect the responses: ‘1’ indicated strong disagreement and ‘5’ indicated strong agreement.

### Data Analysis

Migrant dentists were categorised into three mutually exclusive groups based on their country of primary dental qualification and route towards registration in Australia: Direct Recognition, Examination Pathway (or ADC Successful: The ADC is a four stage examination process involving a general assessment, English language test, preliminary written test, and a practical clinical test) and Alternative Pathway.^7^ Migrants with a primary dental qualification from New Zealand, the United Kingdom, Republic of Ireland, and Canada were classified as Direct Recognition; primary qualifications from these countries were recognised for practice in Australia, without any further examination. Migrant dentists’ having participated and successfully completed the ADC examination process were categorised as ADC Successful. This group of migrant dentists were mainly from the low- and middle-income countries. The Alternative Pathway group included dentists working in the public sector dental workforce scheme, or as academics/researchers/students or specialists or those who migrated to Australia at a time when mutual qualifications from other countries were recognised.

The year of birth was used to derive average age and age groups. The postcode of main practice location was linked with Australian Standard Geographic Classification (ASGC) Remoteness Areas^30^ data in order to provide a new variable relevant to the relative remoteness of practice location.

All the LSE scales were dichotomized into two groups using mean scores less than four as the cut-off point and coded as indicator variables. This approach was based on prior studies that have used a similar cut-off point.^31,32^ Resultant values greater than or equal to 4 were coded as 1 (indicating strong agreement) and values less than 4 were coded as 0 (other). These dichotomised life story scales were then examined by selected characteristics of migrant dentist. Chi-square tests were used with a level of significance set at *P *< .05. Migrant dentist characteristics were entered as covariates in a series of logistic regression models. The dichotomised LSE scales were treated as dependent variables. Entry of variables in the model was conceptually based to establish the most parsimonious model^33^ - fewer terms as possible, but both conceptually relevant and able to explain the predicted LSE of migrant dentists. Adjusted odds ratios were generated. All data was analysed using IBM SPSS version 20.^34^

## Results

Overall, 1022 migrant dentists responded to the national survey, resulting in an response rate of 54.5%.

### Sample Characteristics

Table 1 presents the number and percentage of respondents by selected characteristics of migrant dentists. The Direct Recognition group comprised the largest proportion of migrants (48.0%), followed by the ADC Successful (40.2%) and the Alternative Pathway (11.7%) groups. The majority of participants were male (58.2%), and over half of all participants (53.6%) had children less than 18 years of age. Further, over half of the respondents (51.2%) were aged 45 years or older, and a larger proportion (58.0%) had arrived in Australia 10 or more years ago. Over three-quarter of the participants (76.2%) practised in major cities in Australia, and a large majority reported working in private practices (88.4%).

**Table 1:** Sample Characteristics

**Variable**	**Study Sample**	
	**No.**	**Percent**
Migrant dentist groups (n = 1022)		
Direct Recognition	491	48.0
ADC Successful	411	40.2
Alternative Pathway	120	11.7
Sex (n = 1021)		
Male	594	58.2
Female	427	41.8
Age group (n = 1018)		
Less than 35 years	226	22.2
35 to 44 years	271	26.6
45 to 54 years	237	23.3
55 to 64 years	201	19.7
65+ years	83	8.2
Years since arrival to Australia (n = 885)		
Less than 5 years	158	17.9
5 to 9 years	214	24.2
10 to 19 years	222	25.1
20 to 29 years	139	15.7
30+ years	152	17.2
Children (n = 895)		
Have children under 18 years	480	53.6
Do not have children under 18 years	415	46.4
Remoteness area of main practice (n = 932)		
Major city	710	76.2
Inner regional	148	15.9
Outer regional	65	7.0
Remote/very remote	9	0.9
Type of main practice (n = 921)		
Public	107	11.6
Private	814	88.4

Abbreviation: ADC; Australian Dental Council. Note: Sample characteristics are based on full sample (n) = 1022. Percentages may not add to 100 due to rounding.

### Life Story Experience Scales by Migrant Dentist Characteristics

Table 2 presents bivariate associations between the dichotomised LSE scales and migrant dentist characteristics. The total number of respondents (n) and proportion in agreement with each scale are presented. “Health system and general lifestyle concerns” home country scale (LSE1) varied by migrant dentist groups, sex, age group, years since arrival in Australia and having children less than 18 years of age. Both Australia based LSE scales (LSE2 and LSE3) were associated with migrant dentist groups, sex, age group, and years since arrival to Australia. Further, “appreciation towards Australian way of life” (LSE2) scale varied by type of main practice; “settlement concerns” (LSE3) scale also varied by having children less than 18 years of age. LSE4 (society and culture) home country scale varied by remoteness area of main practice location, and LSE5 (career development) was associated with age group and years since arrival to Australia.

**Table 2:** Bivariate Analysis of the LSE Scales by Sample Characteristics

	**LSE1**		**LSE2**		**LSE3**		**LSE4**		**LSE5**	
	**No.**	**Strongly Agree / Agree (%)**	**No.**	**Strongly Agree / Agree (%)**	**No.**	**Strongly Agree / Agree (%)**	**No.**	**Strongly Agree / Agree (%)**	**No.**	**Strongly Agree / Agree (%)**
Migrant dentist groups		*		*		*				
Directly Recognition	458	1.3	452	60.2	408	15.2	474	51.1	464	41.4
ADC Successful	369	18.2	364	50.8	361	57.6	380	57.4	371	41.5
Alternative Pathway	111	7.2	109	49.5	94	28.7	114	58.8	112	52.7
Sex		*		*		*				
Male	550	7.1	542	58.5	501	31.5	568	52.1	555	44.7
Female	387	10.9	382	50.8	362	38.4	399	57.9	391	40.2
Age group		*		*		*				*
Less than 35 years	205	14.1	204	47.1	190	46.8	213	53.5	210	35.2
35 to 44 years	254	12.6	249	45.8	241	49.8	256	57.8	252	41.3
45 to 54 years	213	4.7	209	63.2	198	22.7	221	54.3	216	44.4
55 to 64 years	185	3.2	184	64.7	162	19.8	192	54.2	185	50.8
65+ years	78	3.8	76	63.2	70	12.9	82	46.3	80	45.0
Years since arrival		*		*		*				*
Less than 5 years	144	8.3	143	37.1	132	39.4	148	55.4	146	50.0
5 to 9 years	200	16.0	196	46.4	193	49.2	204	52.9	201	32.3
10 to 19 years	202	5.0	198	58.6	193	32.1	208	56.7	204	44.6
20 to 29 years	129	4.7	128	65.6	117	24.8	134	55.2	131	48.1
30+ years	138	2.9	137	70.8	118	5.9	145	49.0	138	42.8
Children		*				*				
Have children under 18 years	437	10.5	428	54.9	414	44.4	447	53.7	438	42.0
No children under 18 years	386	5.4	384	57.6	347	25.1	401	54.6	390	45.4
Remoteness area of main practice								*		
Major cty	665	8.0	660	55.0	607	32.3	689	58.1	674	45.0
Inner regional	142	14.1	137	58.4	132	37.9	143	46.9	140	35.7
Outer regional	62	6.5	61	50.8	61	41.0	63	39.7	63	41.3
Remote/very remote	9	11.1	9	77.8	8	12.5	9	55.6	9	33.3
Type of main practice				*						
Public	96	6.3	94	39.4	92	42.4	98	61.2	97	37.1
Private	755	9.3	744	57.5	693	32.3	780	53.6	763	43.3

Abbreviations: LSE, life story experience; LSE1 is Health system and general lifestyle concerns scale; LSE2 is Appreciation towards Australian way of life scale; LSE3 is Settlement concerns scale; LSE4 is Society and culture scale; LSE5 is Career development scale; ADC, Australian Dental Council. Note: Shaded areas represent home country based scales on experiences that contributes to dentist migrating to Australia; Unshaded represent scales based on settlement experiences in Australia. * *P* < .05; Chi-square test.

Logistic regression analysis between the dichotomised LSE scales and migrant dentist characteristics is presented in Table 3. Adjusted models are presented for each scale. Compared with the reference category (Direct Recognition group), the odds ratio (OR) for the ADC Successful group (9.32; 3.51-24.72) and Alternative Pathway group (7.38; 2.04-26.73) were significantly higher for concerns on “health system and general lifestyle concerns” home country scale (LSE1). Both the home country based scales – “society and culture” (LSE4) and “career development” (LSE5) – varied with the remoteness area of main practice. Migrant dentists practising in the rest of state (ie, regional and remote areas) had lesser affinity towards home country’s society and culture (LSE4: 0.59; 0.40-0.85) and appeared to have received fewer career development opportunities in home country (LSE5: 0.64; 0.43-0.94), when compared to the reference category (major city). Further, migrant dentists aged 55+ years old reported to have received better career development opportunities in their home countries (LSE5: 2.15; 1.06-4.38), when compared to the reference category (less than 35 years old).

**Table 3:** Logistic Regression Analysis (Adjusted Model) of the LSE Scales by Sample Characteristics

		**LSE1**			**LSE2**			**LSE3**			**LSE4**			**LSE5**	
		**OR**	**95% CI**		**OR**	**95% CI**		**OR**	**95% CI**		**OR**	**95% CI**			**OR**	**95% CI**	
Migrant dentist groups																				
Directly Recognition		Ref.				Ref.				Ref.				Ref.				Ref.		
ADC Successful	**	9.32	3.51	24.72	*	0.66	0.46	0.96	**	5.39	3.51	8.28		1.36	0.95	1.94		0.94	0.65	1.35
Alternative Pathway	**	7.38	2.04	26.73		0.83	0.48	1.43		1.88	0.91	3.90		1.63	0.96	2.74		1.45	0.86	2.44
Sex																				
Male		Ref.				Ref.				Ref.				Ref.				Ref.		
Female		1.28	0.67	2.42		0.76	0.53	1.10		0.84	0.56	1.28		1.30	0.92	1.83		1.04	0.73	1.48
Age group																				
Less than 35 years		Ref.				Ref.				Ref.				Ref.				Ref.		
35 to 54 years		0.96	0.44	2.10		0.64	0.38	1.09		0.66	0.36	1.20		0.83	0.50	1.39		1.28	0.76	2.17
55+ years		0.66	0.14	3.18		0.59	0.29	1.22		0.59	0.25	1.37		0.87	0.43	1.75	*	2.15	1.06	4.38
Years since arrival to Australia																				
Less than 10 years		Ref.				Ref.				Ref.				Ref.				Ref.		
10 to 29 years	**	0.32	0.14	0.74	**	1.97	1.27	3.05		0.63	0.39	1.02		1.14	0.74	1.74		0.92	0.60	1.41
30+ years		0.32	0.04	2.34	**	2.90	1.40	5.99	*	0.23	0.07	0.74		0.98	0.49	1.95		0.54	0.27	1.08
Children																				
Have children under 18 years		Ref.				Ref.				Ref.				Ref.				Ref.		
No children under 18 years		0.87	0.42	1.81		0.79	0.53	1.18	**	0.50	0.31	0.80		1.07	0.73	1.56		0.95	0.65	1.38
Remoteness area of main practice																				
Major city		Ref.				Ref.				Ref.				Ref.				Ref.		
Rest of state		1.37	0.70	2.68		1.19	0.80	1.78		0.977	0.61	1.56	**	0.59	0.40	0.85	*	0.64	0.43	0.94
Type of main practice																				
Public		Ref.				Ref.				Ref.				Ref.				Ref.		
Private		1.84	0.61	5.55	**	2.31	1.31	4.06		0.87	0.47	1.64		0.75	0.44	1.28		1.05	0.62	1.79

Abbreviations: LSE, life story experience; LSE1 is Health system and general lifestyle concerns scale; LSE2 is Appreciation towards Australian way of life scale; LSE3 is Settlement concerns scale; LSE4 is Society and culture scale; LSE5 is Career development scale; ADC, Australian Dental Council; OR, odds ratio. Note: Shaded areas represent home country based scales on experiences that contributes to dentist migrating to Australia; Unshaded represent scales based on settlement experiences in Australia. *P < .05, **P < .01.

The ADC Successful group reported less appreciation towards the Australian way of life (LSE2: 0.66; 0.46-0.96), when compared to Direct Recognition group. The appreciation towards Australian way of life improved with age - compared to the reference category of less than 10 years, being in Australia for 10 to 29 years, and 30+ years both had a higher odds ratio (1.97; 1.27-3.05 and 2.90; 1.40-5.99, respectively). Further, migrant dentists working in private practice have reported greater appreciation towards the Australian way of life (LSE2: 2.31; 1.31-4.06), when compared to the reference category, public dental practice. The ADC Successful group also appeared to face greater “settlement concerns” in Australia, (LSE3: 5.39; 3.51-8.38) in comparison with the reference category, Direct Recognition group. Settlement concerns also seemed to reduce with years since arrival to Australia, and the 30+ year group had the lowest odds ratio (0.23; 0.07-0.74). Further, migrant dentists having no children under 18 years old appeared to have faced fewer settlement concerns (0.50; 0.31-0.80), when compared to the reference category of having children under 18 years old.

## Discussion

The purpose of the study was to assess the variation between migrant dentist experiences and migrant dentist characteristics in Australia, in order assist policy challenges facing dentist migration. The findings offer some suggestions that migrant dentists from developing countries face greater challenges both in their home countries and in Australia, compared to migrant dentists from well-developed countries.

### Life Story Experience Scales

The study used ‘natively’ developed LSE scales. The scales included five experience domains: three based on home country events and two on settlement experiences in Australia. As the scales were designed through a grounded qualitative approach, including the actual narrations of migrant dentists, it is likely to reflect sentiments of migrant dentists in Australia.^29^ Further, being based on actual migrant dentist narrations, the results can offer insights to policy-makers and support future research on migrant dentists.^29^ The five LSE domains were intended to be assessed separately, so it could offer in-depth information on the experiences associated with each domain; a single composite measure of LSE was not essential or informative. The interpretation of results to each of the five LSE domains also required attention. The “health system and general lifestyle concerns” home country scale and “settlement concerns in Australia” scale brought out concerns or misgivings experienced by migrant dentists; other scales (Australian way of life, society, and career related issues at home country) brought out appreciation or affinity towards the underlying concept of each of the scales.

### Health System and General Lifestyle Concerns in Home Country

The adjusted logistic regression model for the “health system and general lifestyle concerns” home country scale (LSE1) found associations that were prominent only for migrant dentist groups and years since arrival to Australia. The ADC Successful and Alternative Pathway group of migrant dentists were more likely to have experienced difficulties and were less satisfied with home country systems and living conditions, compared with the Direct Recognition group (from well-developed countries). A vast majority of participants from the ADC Successful and Alternative Pathway groups were from developing countries, showing major shortcomings in dental workforce policy and planning.^7^ Countries such as India,^15,16^ Philippines,^35,36^ Thailand,^18^ and Indonesia^17^ have dramatically upscaled the production of dentists, yet with little consideration towards the organization and delivery of dental services.^37,38^ Much of the contribution to this increase in dentists is from the recent increase in private dental colleges. Dentists from many developing countries are also involved in private practices^37^; the rise in the number of dentists has increased competition in the metropolitan areas that appear already well-supplied with dentists.^39^ Public dental services and rural areas are the most affected.^37^ Dental workforce planning and migration policies in these countries will require to first understand the underlying complexity of the dentist migration issue, the range of factors contributing towards migration and their influence on health service delivery. For example, addressing the emigration of dentists by seeking to produce more dentists might have little improvement on overall health service delivery due to the intrinsic deficiencies that exist in the health systems in sending countries.

Broader issues such as corruption and poor living conditions also seem to coexist with health system deficiencies and affect dentist experiences. Some countries in the African and Middle Eastern regions have been experiencing unstable political environments and more complex issues such as racism and discrimination.^40,41^ Dentists seem to migrate, as they feel disappointed by home country systems.^1^ Further, many countries in the poorer regions of the world do not have suitable workforce surveillance systems, and very little is known on the migration flows of dentists in order to support policy-making decisions.^37^ Similar concerns also exist in physician and nurse migration.^42-44^ While integration of dentist migration policies with the broader health professional migration is a logical approach, policies developed to address health professional migration as a whole will also need to take into account the differences in oral healthcare systems (such as predominant private practices).

### Migrant Dentist Experiences in Australia

The ADC Successful group of migrant dentists (mainly from developing countries) appear to have a lesser “appreciation towards the Australian way of life” scale (LSE2), and have experienced more settlement concerns (LSE3) in Australia. Migrant dentists involved in the qualifying examination process have expressed sentiments on the tough and stressful nature of the examination, and the impact of the examination on one’s finances and time. This study supports prior qualitative studies that called for support structures for migrant dentists involved in the examination process.^14,20^ Since 2014, a new assessment and examination process has been introduced by the ADC^14^ that includes a shorter clinical assessment process.^13^ However, further research is required to understand the impact of recent changes towards migrant dentist experiences.

Migrant dentists are more appreciative towards life in Australia if they have lived longer in Australia. Our finding provides new evidence to an argument that current policies and programs directed at recent migrants to facilitate their integration to the Australian way of life could be ineffective, and need reconsideration. Currently, support for migrants is offered by immigration departments or more specifically by universities and the public dental sector.^19^ A large proportion of migrant dentists might not avail themselves of these services. Migrants might face broader challenges such as seeking work or improving their financial position or settling down with a family – therefore, gaining an understanding towards the Australian way of life might not be an immediate priority. Prior research has suggested the role of friends and family in improving the cultural adaptation process of migrant dentists in Australia.^19^ Strengthening support structures for migrant dentists (and other migrant healthcare professionals) through direct involvement of local networks and cultural groups is a viable alternative to facilitate integration of migrant dentists within the Australian community.

Migrants also appear to appreciate work in the private dental sector. The limitations of public sector in terms of scope of practice of a dentist,^27^ and/or better financial returns of working in the private sector, could have contributed towards migrant dentist experiences in Australia. In order to circumvent the limited scope of practice in the public sector, it would be essential to offer better professional development and career opportunities for migrant dentists working in the public sector clinics. Further research in this direction is recommended.

### Affinity Towards Home Country

In the adjusted analysis, participants living in non-metropolitan areas were less likely to have expressed affinity to home country “society and culture” scale (LSE4). Prior studies lend support to an argument that migrants closely knit to family and friends, are more likely to be socially active,^1,45^ and possibly aim to preserve such culture. While there is not enough evidence to suggest that this attraction will make emigration to home countries imminent, we can at least argue that such migrants will tend to live in areas where they can experience closeness to their native cultures. Metropolitan areas in Australia have vibrant multicultural hubs, providing a similar community experience.^46^ More attention is required towards migrant dentists living in non-metropolitan areas gain better understanding of their cultural adherence, and assimilation of Australian values.

Younger migrant dentists in Australia were less likely to be in agreement with the “career development” in home country scale (LSE5). The dental education systems in some developing countries (eg, India and Philippines) are producing surplus graduates with a possible intention to make them available to a global market.^37,47^ Current dental education is more technology-driven and less problem-centric.^48^ As a consequence, migrant dentists may not realise the potential for career development in home countries and have a desire to practice high-end dentistry.^1^ Policies that address dentist migration need also to focus on the emerging workforce, so as to cultivate new dental education and practice philosophies that are in line with the needs of the local population.

### Health Workforce Governance and International Cooperation

In the recent years, there is a growing international consensus on integrated health workforce governance, and the urgent need for better attention to health workforce issues both in the national and global agendas.^49-51^ Migration of health personnel (including dentists) has contributed to health workforce crisis in several countries.^43,51^ As the migration issue spreads across more than one country, the concept of global/regional planning makes sense.^43^ The LSEs of migrant dentists in Australia, provides evidence to support a multi-level health workforce governance. The crux of this approach is to develop evidence at a global (or) regional level that can potentially contribute towards national level workforce planning and migration policies. This approach could enable developing and poorer regions in the world to avail the combined expertise of a ‘group’ of (both sending and receiving) countries in order to tackle migration, and improve health workforce planning. However, the pathway to achieve multi-level health workforce governance will first require support from participating countries and consensus to integrate dentist migration to the mainstream health professional migration. Some contemporary examples of multilevel approaches, though in early stages, include the European Union (EU),^51,52^ Gulf Coopeartive Council,^53^ and Association of South East Asian Nations.^54^

The World Health Organization (WHO) global code of international recruitment of health personnel (WHO code) is recognised as a core component of bilateral, national, regional, and global responses to the challenges of health personnel migration and health systems strengthening.^55,56^ Articles 4-10 of the WHO code provides a detailed framework for dialogue and international cooperation among national and international stakeholders.^55^ This includes guidelines for recruitment, advocacy for domestic workforce planning, improving data gathering, research and information exchange, importance of partnerships between state and non-stateplayers, and improved technical collaboration.^57^ This study supports the WHO code and recognises the significance of health workforce governance approaches to evolve or include the WHO code as a fundamental aspect of migration policies.

## Limitations

The sampling frame for the study was based on migrant dentist registrations in the Australian Dental Association and enrolment information provided by the participating dental schools.^7^ A more exhaustive list of migrant dentists, based on the national register of dentists,^58^ was not available for research purposes. Nevertheless, over 90% of all practising dentists in Australia are also members of the dental association.^59^ The inclusion of graduate students in dental schools improved representativeness of the study sample.^7^ We did not survey migrants who were involved in a non-dental job (or) non-dental university study program (or) who were unemployed. The identification of these migrant dentists would require integration of several data sources such as immigration, dentist registration, dental association membership, student enrolment and possibly taxation and social service information.^7,60^ Linkage between these systems is not in place in Australia. The study included migrant dentists, who were residing in Australia at the time of the survey. It is possible that migrants living in Australia could exhibit a different set of experiences compared to those who have left Australia. Prior studies in general population movement offer various explanations^61-63^; ‘return migration’ to home country or emigration to a third country is more common among migrants who have experienced severe failure or among the more successful migrants who have accumulated considerable financial wealth. In order to include all migrant experiences (those who emigrate or returned), would require an international effort first addressing challenges in migrant dentist immigration and surveillance system.^60^ Future studies could benefit from a multicountry approach to better account for global mobility, so as to offer a more detailed assessment on the experiences of the mobile migrant dental workforce.

## Conclusion

Migrant dentists from developing countries (mainly the ADC Successful group) face broader challenges, compared to other migrant dentist groups, both in their country of origin and in Australia. Migrants appear to be let down by home country systems and have reported shortcomings in the structure, organization, and delivery of dental services. Broader issues such as corruption and poor living conditions coexist with health system deficiencies and influence dentist experiences. Migrant dentists, who have migrated to Australia more recently, and migrants with children, have experienced greater settlement concerns in Australia. Migrants appear to value working in the private sector. This study offers recommendations towards targeted policies for migrants facing settlement concerns in Australia. A further suggestion is to adopt a multi-level health workforce governance approach and call for greater consensus towards an international agenda to address the dentist migration issue. This global strategy should assist both developed and developing countries understand the dentist migration problem, and support in dental workforce planning and migration policy.

## Acknowledgments

The first author was supported by an Australian Postgraduate Research Scholarship during the time the fieldwork and analysis were conducted and later support from an NHMRC’s Centre for Research Excellence in Health Services Research (No. 1031310), and an Endeavour Fellowship from the Australian Government Department of Education and Training. This study was also supported by a grant from the Australian Dental Research Foundation (64-2011). We are grateful for the assistance offered from colleagues in the Australian Dental Association Inc. (Federal Branch), ADC and the Australasian Council of Dental Schools (ACODS) for assistance in the fieldwork. The contents are solely the responsibility of the administering institution and authors, and do not reflect the views of NHMRC.

## Ethical issues

Ethical approval for the study was obtained from the Human Research Ethics Committee of the University of Adelaide, Adelaide, SA, Australia, and was conducted in accordance to the Declaration of Helsinki. The survey was administered as mailed self-complete questionnaire; consent was, therefore, implied by the return of the completed questionnaire.

## Competing interests

Authors declare that they have no competing interests.

## Authors’ contributions

MB designed the study, conducted fieldwork, collected data, supervised data entry, performed analysis, and wrote the manuscript. DSB supervised the development and progress of the study; contributed to the study design, and overall analysis of the study; drove the funding and ethics applications; provided intellectual content and revised the manuscript. AJS and SDS both supervised the development of the study, contributed to the study design, provided intellectual content, and revised the manuscript. Further, AJS also provided inputs towards the overall strategy for the study. KW provided expert opinion towards the revision of the manuscript. SC provided expert opinion towards statistics analysis, data linkage, and the contributed towards the revision of the manuscript.

## Authors’ Affiliations

^1^Australian Research Centre for Population Oral Health, School of Dentistry, The University of Adelaide, Adelaide, SA, Australia. ^2^Discipline of Behavioural and Social Sciences in Health, Faculty of Health Sciences, The University of Sydney, Sydney, NSW, Australia. ^3^Australian Dental Council, Melbourne, VIC, Australia.

## Supplementary Files

Supplementary file 1 Supplementary file 1 contains Table S1.

## Key messages

**Implications for policy makers**

- The study provides evidence to support a multi-level health workforce governance. The crux of this strategy will be to strengthen evidence at a global (or) regional level that could offer support to national level workforce policies.
- Better integration of dentist migration (and dental workforce governance) with the mainstream health workforce governance is a viable and opportunistic way forward.

**Implications for public**

Migrant dentists appear to be let down by home country systems and have reported shortcomings in the structure, organization, and delivery of dental services. Broader issues such as corruption and poor living conditions seem to coexist with health system deficiencies and influence migrant dentist experiences. Dentists who have migrated to Australia more recently, and migrants with children, have experienced greater settlement concerns in Australia. This study offers recommendations towards targeted policies for migrant dentists facing settlement concerns in Australia. A further suggestion is to adopt a multi-level health workforce governance approach and call for greater consensus towards an international agenda to address the dentist migration issue.
