# Supplementary material for: The Life Story Experience of "Migrant Dentists" in Australia: Potential Implications for Health Workforce Governance and International Cooperation
Source: Int J Health Policy Manag. 2016 Oct 10;6(6):317–26. doi: 10.15171/ijhpm.2016.135 (PMC5458793; doi:10.15171/ijhpm.2016.135)
Supplement: Supplementary file 1 — contains Table S1. [file ijhpm-6-317-s001.pdf]

IJHPM
International Journal of Health Policy and Management

Table S1. List of Items in the LSE Scales

Scale/Item	Description of Scale/Item
**LSE1**	**Health system and general lifestyle concerns**
1	I was affected by corruption in my day to day practice life in my home country.
2	I thought the health service infrastructure in my home country was very good. *
3	Patients did not receive quality care in public clinics/hospitals back home.
4	There was too much competition between dentists in my home country.
5	Dentistry was not seen as a priority for policy-makers in my home country.
6	The quality of dental practice was not good in my home country.
7	There are too many dental colleges/schools in my home country.
8	The oral health service system in my home country needs improvement.
9	The living standards in Australia are better than my home country.
10	Australia is somewhat similar to my home country. *
**LSE2**	**Appreciation towards Australian way of life**
11	I like the cultural diversity in Australia.
12	Australians have been very kind to me.
13	I found it easy to settle down in Australia.
14	I haven't felt any issues of discrimination in Australia.
15	I have made very good friends here in Australia.
16	The quality of life is better in Australia.
17	Australia is a safe place to live.
18	The standards of dentistry in Australia are very high.
19	Professional life in Australia is enjoyable.
20	Working in private practice is enjoyable.
21	There are good professional development opportunities in Australia.
22	I find myself very comfortable in the place I am staying right now in Australia.
23	I love Australia very much.
**LSE3**	**Settlement concerns**
24	The ADC exam process is very long.
25	Support structures to prepare for the exam are very important.
26	It takes lot of hard work to start a private practice in Australia.
27	Specialist registration requirements in Australia are difficult.
28	In future, I would like to see myself a bit higher in professional status.
29	I want my children to understand the culture of my home country.
30	I am planning to spend more time with my family in the future.
**LSE4**	**Society and culture**
31	I had a very active social life in my home country
32	I come from a tight-knit family.
33	I loved the lifestyle back in my home country.
34	I did not have enough time for social activities in my home country. *
**LSE5**	**Career development**
35	I felt I had good hand skills, so I opted for dentistry.
36	I was very happy with my professional career in my home country.
37	I had very good mentors in my home country.
38	I had adequate professional development opportunities in my home country.

Abbreviations: LSE, life story experience; ADC, Australian Dental Council.

Note: Shaded areas represent home country based scales on experiences that contributes to dentist migrating to Australia; Unshaded areas represent scales based on settlement experiences in Australia; * Negatively worded items.
